# Editorial: Prenatal environmental and genetic interactions: an exploration from fetal development to child health

**DOI:** 10.3389/fped.2025.1753612

**Published:** 2025-12-16

**Authors:** Yalan Li, Tianjiao Liu, Xijing Liu, Jiamin Wang, Ting Hu, Xue Xiao, Xin Li

**Affiliations:** 1 Department of Psychiatry, West China Second University Hospital, Sichuan University, Chengdu, Sichuan, China; 2 Key Laboratory of Birth Defects and Related Diseases of Women and Children, Ministry of Education, West China Second Hospital, Sichuan University, Chengdu, China; 3 Chengdu Women’s and Children’s Central Hospital, School of Medicine, University of Electronic Science and Technology of China, Chengdu, China; 4 Department of Molecular Genetics, Graduate School of Medicine, Kyoto University, Kyoto, Japan; 5 Department of Medical Genetics, West China Second University Hospital, Sichuan University, Chengdu, Sichuan, China; 6 Department of Obstetrics, West China Second University Hospital, Sichuan University, Chengdu, Sichuan, China

**Keywords:** fetal development, child health, genetic, maternal-fetal medicine, environment

The idea that the intrauterine environment can shape lifelong health has increasingly been recognized as an important theme in contemporary pediatrics, perinatal medicine, and developmental science. The Developmental Origins of Health and Disease (DOHaD) paradigm proposes that pregnancy is not merely a transient physiological state, but a critical window during which environmental signals “program” trajectories of growth, metabolism, neurodevelopment, and disease susceptibility (1, 2). Within this framework, the fetus is viewed as an active responder to a complex milieu of nutritional, hormonal, inflammatory, and toxic exposures that may have consequences extending well beyond birth (3).

In this context, prenatal environmental factors and genetic or epigenetic influences can no longer be considered in isolation. Maternal nutrition, cardiometabolic status, tobacco smoke, heavy metals, infections, and psychosocial stress interact with inherited variation and dynamic epigenetic marks to shape organogenesis, immune maturation, endocrine regulation, and brain development (4–6). At the same time, advances in genomics, epigenomics, imaging, and biostatistics have begun to enable a shift from simple associations toward more mechanistic understanding, risk stratification, and the design of early interventions (7, 8). Nevertheless, important knowledge gaps remain regarding how these multiple layers of influence converge in diverse real-world settings and populations.

Against this backdrop, the Research Topic “Prenatal Environmental and Genetic Interactions: An Exploration from Fetal Development to Child Health” was established as a platform to bring together clinical, epidemiologic, mechanistic, and consensus work in this evolving field. The 16 contributions span rare imprinting disorders and placental pathology, common obstetric complications, environmental and nutritional exposures, oral and constitutional health, and the lived experience of families raising children with congenital conditions. Taken together, these articles suggest that improved understanding of prenatal environmental-genetic interactions is an important prerequisite for refining prevention strategies and moving toward more personalized pediatric care.

A first group of studies focuses on common maternal exposures and nutritional factors that are linked to measurable pediatric outcomes. The reported association between prenatal environmental tobacco smoke exposure and reduced selenium concentrations in childhood suggests that a single pervasive exposure, namely tobacco smoke, may disrupt micronutrient status long after birth and potentially compromise antioxidant defenses and immune function. Complementing this, a retrospective clinical study on combined calcium and vitamin D3 supplementation during pregnancy indicates that relatively simple nutritional interventions may be associated with a lower risk of gestational diabetes, preeclampsia, and gestational hypertension, as well as improved birth weight and early neonatal adaptation. Considered together, these findings illustrate both risk and potential resilience: adverse exposures such as tobacco smoke may deplete micronutrient reserves, whereas targeted supplementation may partially mitigate pregnancy-related complications and support early growth.

Several contributions further characterize how maternal cardiometabolic health may influence fetal growth and later outcomes. A longitudinal analysis of three timepoint maternal blood pressure trajectories reports that women with high-medium systolic blood pressure patterns have a substantially increased risk of delivering low birth weight infants, particularly when additional vulnerabilities such as older age, high BMI, low income, or dyslipidemia are present. Another multicenter cohort examining gestational diabetes mellitus (GDM) finds that *in utero* exposure to GDM is associated with higher weight-for-age z-scores at six months and an elevated risk of food allergy, thereby linking the maternal glycemic milieu not only to growth but also to early immune-related outcomes. These studies collectively support the view that it may be useful to move beyond single-measure “snapshot” assessments toward evaluation of dynamic trajectories of blood pressure and glucose, and to consider GDM and hypertensive disorders as early-life programming events with implications for both somatic and immune health in offspring.

Environmental adversity and toxicant exposure constitute another key dimension of prenatal and early-life risk. A nested case-control study from a Chinese birth cohort reports that higher maternal blood concentrations of barium and arsenic, both individually and in combination, are associated with increased odds of congenital heart defects and specific subtypes, with evidence of a multiplicative interaction between the two metals. At the population level, a national survey-based analysis of early-life famine exposure indicates that fetal and childhood exposure to severe undernutrition is associated with a higher risk of physical disability in later life, with nuanced differences by sex, place of residence, and famine severity. Viewed together, these contributions suggest that both acute toxicants and large-scale nutritional shocks can act as powerful prenatal and early-life stressors that leave durable marks on cardiovascular, musculoskeletal, and functional outcomes into older adulthood.

The Topic also includes mechanistic and rare-disease insights that illustrate how genetic and epigenetic factors may intersect with the intrauterine environment. A case report of Beckwith-Wiedemann syndrome with gain of methylation at IC1 and reduced H19 expression highlights the diagnostic value of methylation testing in infants presenting with macrosomia, macroglossia, and umbilical hernia, and underscores the need for long-term tumor surveillance and cardiac monitoring in such cases. At the level of common obstetric complications, a small RNA sequencing study in intrahepatic cholestasis of pregnancy identifies differentially expressed tRNA-derived fragments in maternal serum, with pathway analyses implicating fatty acid degradation. These preliminary data raise the possibility that tRF dysregulation contributes to cholestatic pathophysiology and provide an initial basis for the exploration of novel biomarkers and therapeutic targets. A case report and literature review of placental chorioangiomatosis further draws attention to under-recognized placental vascular lesions that may substantially influence maternal-fetal outcomes and for which prenatal diagnosis, etiologic understanding, and management strategies remain limited.

Bridging basic science, imaging, and clinical practice, an expert consensus on fetal ventriculomegaly provides graded, evidence-based recommendations across 23 key clinical questions spanning diagnosis, etiologic evaluation, antenatal management, delivery planning, and neurodevelopmental follow-up. The consensus emphasizes standardized ultrasound and fetal MRI protocols, routine use of chromosomal microarray analysis in all ventriculomegaly cases, and multidisciplinary decision-making informed by genetics, neonatology, neurology, rehabilitation, and informatics. Importantly, it also highlights the potential future integration of dynamic imaging parameters and multi-omics approaches to refine risk stratification. This work exemplifies how the field is beginning to promote closer integration of technology, genetics, and clinical care.

The downstream expression of prenatal and early-life influences is not limited to organ-specific morbidity. It also appears in oral health and constitutional patterns in early childhood. A cross-sectional study from Shaanxi Province reports that hypomineralized second primary molars affect approximately 7% of 3- to 5-year-old children and are associated with maternal vomiting during pregnancy and lower maternal education. This work suggests that pregnancy-related factors may interfere with enamel mineralization and also points to socio-educational inequities that can exacerbate pediatric oral health disparities. In parallel, the development and validation of a Traditional Chinese Medicine constitution scale for children aged 3–6 years offers an innovative tool to assess physical and mental health profiles in early childhood. With strong internal consistency and acceptable test-retest reliability, this 47-item instrument opens possibilities for integrating constitutional assessment into large-scale health surveys and for exploring how prenatal and early-life exposures may shape holistic health patterns within a specific cultural framework.

Finally, this Topic highlights that the consequences of prenatal and congenital conditions unfold within families and social systems. A cross-sectional survey of caregivers of children with hemifacial microsomia in Chinese online support communities describes substantial medical, financial, and psychosocial burdens. Caregivers, predominantly mothers, report high levels of financial strain, dissatisfaction with surgical outcomes in a subset of children, and heightened sensitivity to social reactions, with rural residence and unemployment further intensifying the care burden. These findings extend the concept of developmental origins beyond biological processes to include the lived experience of families navigating complex congenital conditions, and they emphasize the need to consider integrated medical, psychosocial, and educational support.

Taken together, the articles in this Research Topic span a continuum from molecular signatures and rare imprinting disorders to common obstetric complications, environmental toxicants, nutritional interventions, oral and constitutional health, and family-level psychosocial impacts. Several cross-cutting themes emerge. First, prenatal and very early-life exposures, whether nutritional, metabolic, vascular, or toxic, are repeatedly associated with long-term differences in structural anomalies, growth patterns, functional capacities, and micronutrient status. Second, genetic and epigenetic mechanisms, including imprinting defects and non-coding RNA dysregulation, appear to be key mediators of these associations and will require larger and more integrative studies to be clarified. Third, the importance of robust measurement, whether through blood pressure trajectories, exposure scales, constitution indices, or standardized imaging and genetic protocols, is evident across these studies. Finally, social determinants such as maternal education, income, and rural-urban residence consistently modulate risk and resilience, suggesting that efforts to advance precision child health should be informed by both biological data and equity considerations.

Looking ahead, future research inspired by this Topic may usefully prioritize longitudinal, multi-omics cohorts that link detailed prenatal exposure profiling with genetic, epigenetic, and imaging data, and extend follow-up into childhood and adulthood (9). Integrating mechanistic insights with scalable screening tools and pragmatic interventions, such as micronutrient supplementation, optimized cardiometabolic management in pregnancy, and targeted smoking cessation support, is likely to be important for translating DOHaD principles into clinical practice and public health policy (10). Equally important will be the systematic incorporation of family-centered and psychosocial perspectives, so that interventions address not only biological risk but also the real-world contexts in which children grow and develop.

On behalf of the Topic Editors, we express our sincere appreciation to all authors, reviewers, and multidisciplinary collaborators who contributed to this Research Topic. Their collective work adds to the growing evidence on how prenatal environmental and genetic interactions may shape the foundations of child health and provides a valuable starting point for future research and preventive strategies.
